# Large ganglioneuroma presenting as presacral mass

**DOI:** 10.1259/bjrcr.20150361

**Published:** 2016-11-02

**Authors:** Nagaveni Kamatam, Edward Rayappan, Samjee Smile, Ravichandran Vivekanandan

**Affiliations:** Radio diagnosis, Indira Gandhi Government General Hospital and Post Graduate Institute, Pondicherry, India

## Abstract

Ganglioneuromas are benign tumours of the sympathetic nervous system that originate from neural crest cells. They are extremely rare in the presacral region. Here, we report a case of a presacral mass in a 14-year-old female who presented with complaints of pelvic discomfort, difficulty with micturition and constipation. Ultrasonogram showed a large heteroechoic solid mass in the pelvis with bilateral hydroureteronephrosis. It appeared as a well-circumscribed, hypodense mass lesion on CT scan, measuring 14 x 11 x 10 cm. It appeared isointense to muscle on *T*_1_ and heterogeneously hyperintense on *T*_2_ weighted images with heterogeneous post-contrast enhancement on MRI. The lesion was seen in the presacral region displacing the rectum and bladder anteriorly, and extending posteriorly, causing widening of the sacral foramina. The mass was surgically excised and her symptoms resolved. Histopathological examination of the mass revealed features of ganglioneuroma. We report this case in view of the large size of the mass and its rare location.

## Case history

A 14-year-old female presented with a 9-month history of pelvic discomfort, difficulty with micturition and constipation. She was referred for abdominal ultrasonography, which showed a large heteroechoic solid mass in the pelvis with bilateral hydroureteronephrosis. Bladder, uterus, ovaries and rectum were seen separately, with anterior displacement of these structures. A possibility of teratoma/neurogenic tumour was suspected as the mass was seen in the presacral region.

**Figure 1.**
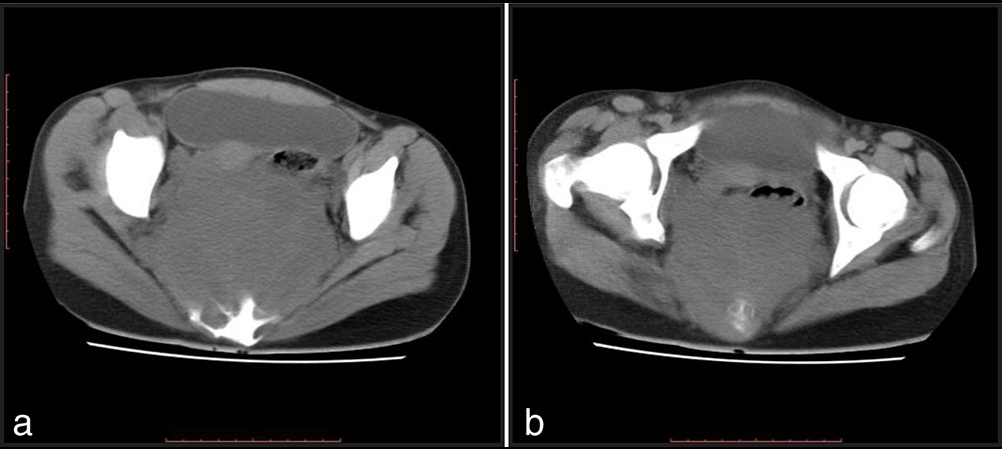
(a, b) Axial CT images in soft tissue window showing a homogeneous mass in the pelvis displacing the rectosigmoid and uterus anteriorly, and compressing the bladder.

**Figure 2.**
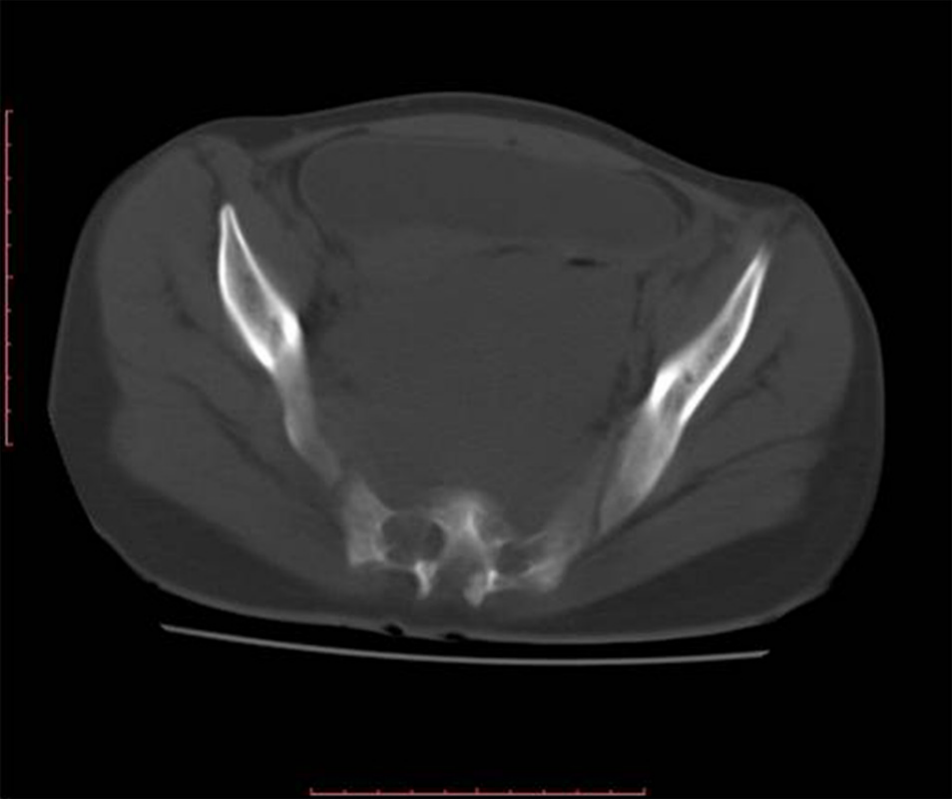
Axial CT image in the bone window showing widening of the sacral foramina.

CT scan of the abdomen and pelvis was performed. A well-circumscribed, homogeneous, hypodense mass lesion was seen in the presacral region of the pelvis. The bladder, uterus and rectosigmoid colon were displaced anteriorly by the mass (Figure 1). The mass measured 14 × 11 × 10 cm; it was seen distorting the sacrum and widening the sacral foramina (Figure 2). No calcifications or cystic areas were seen within. The most likely diagnosis was that of neurogenic tumour.

**Figure 3.**
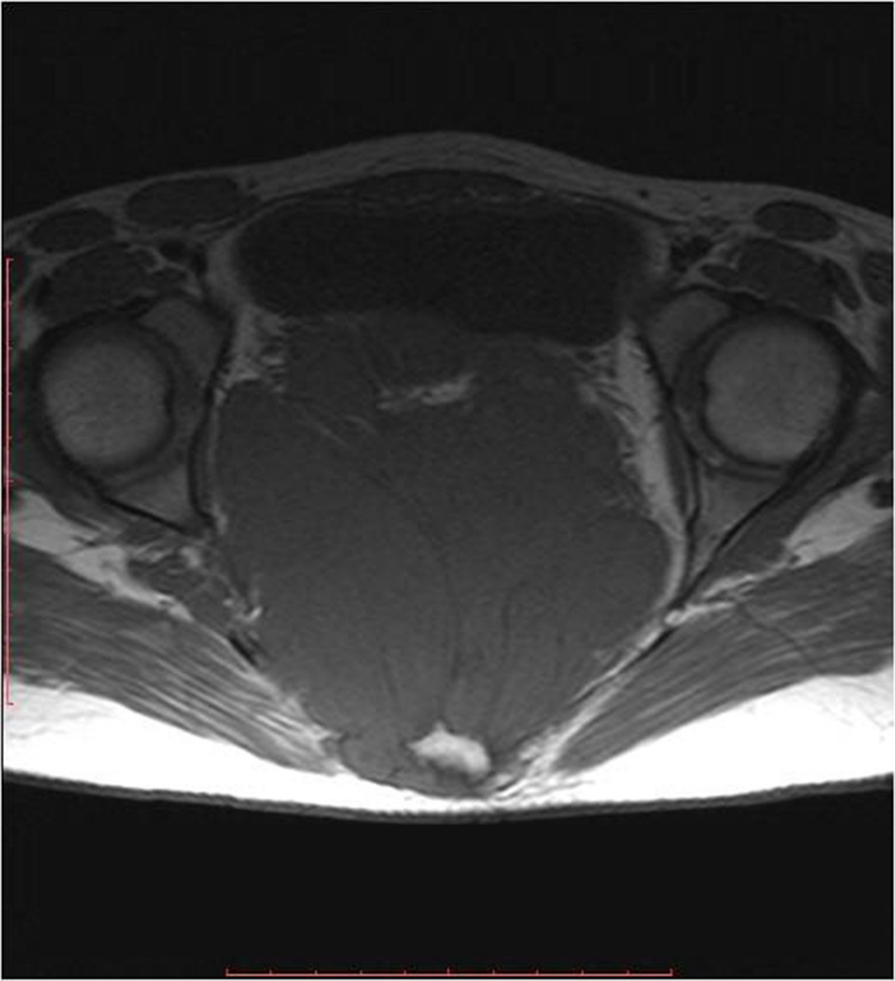
Axial *T*_1_ weighted images showing a hypointense mass lesion with linear hypointense areas.

**Figure 4.**
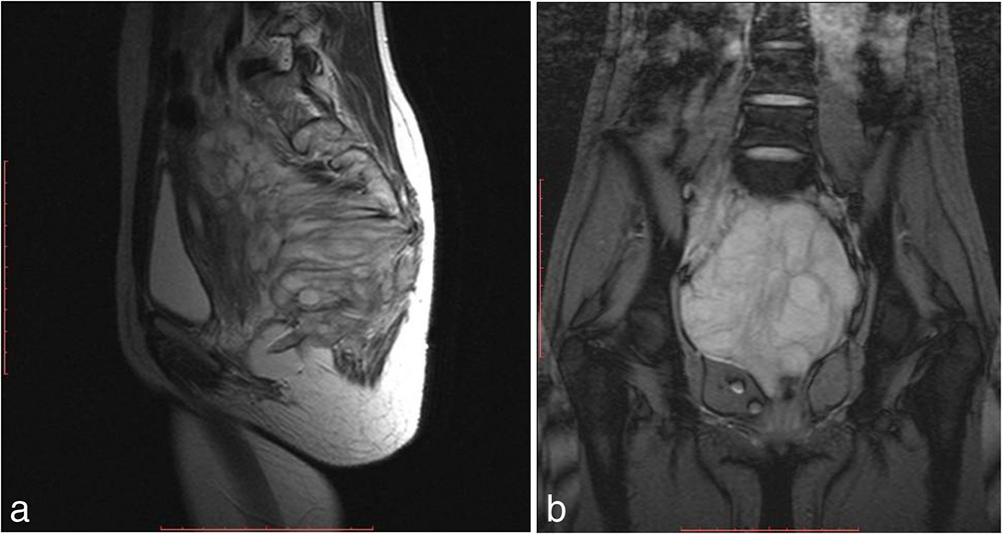
(a) Sagittal *T*_2_ weighted image showing a heterogeneously hyperintense mass in the sacral region extending posteriorly through the sacral foramina. (b) Coronal gradient recalled echo image showing hypointense linear areas within the mass.

**Figure 5.**
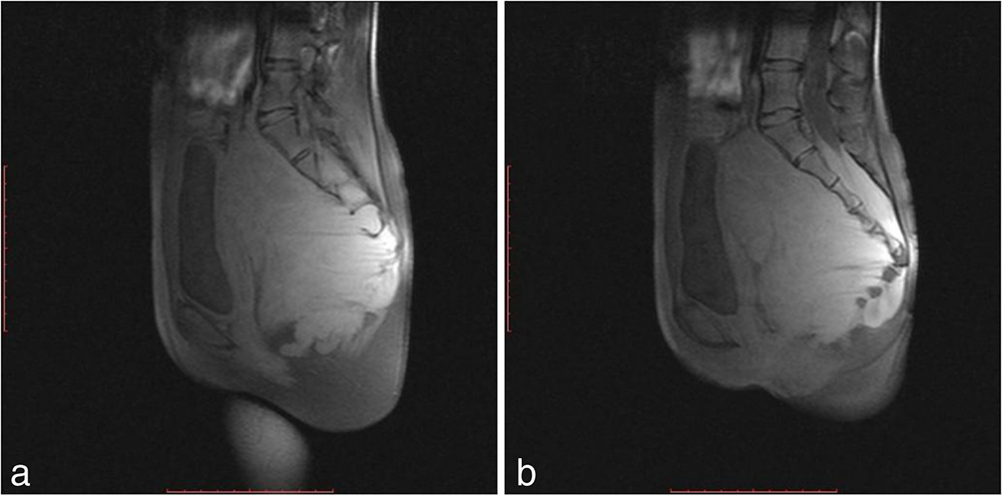
(a, b) Sagittal *T*_1_ fat-suppressed images showing distortion of sacrococcyx, with linear hypointense bands seen coming from the neural foramina.

**Figure 6.**
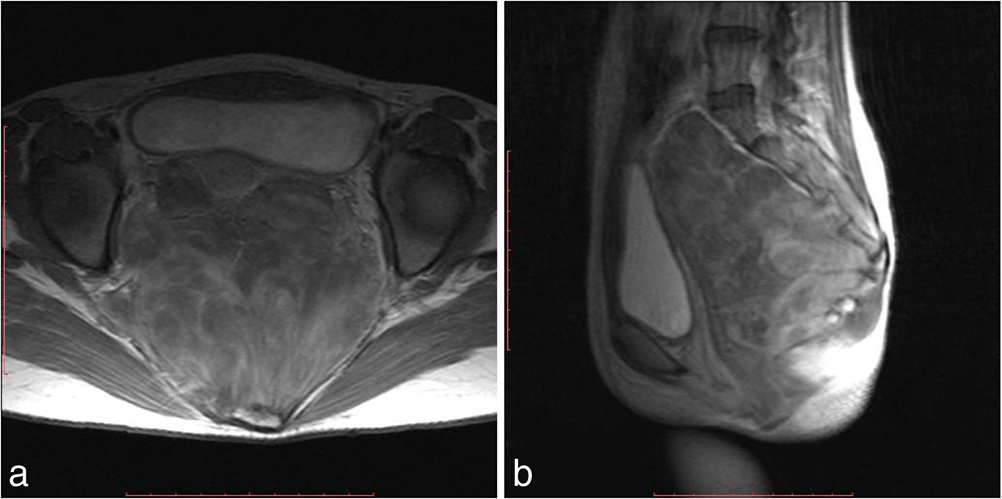
(a) Axial and (b) sagittal post-gadolinium images showing heterogeneous enhancement of the mass.

MRI of abdomen and pelvis was performed using the standard protocol. The mass appeared isointense to muscle on *T*_1 _ and heterogeneously hyperintense on *T*_2_ weighted images. The mass was seen extending posteriorly to the gluteal region, with widening of multiple sacral foramina. No cystic or haemorrhagic areas were noted. On *T*_2_ weighted images, linear and wavy hypointense areas were noted within the mass, suggesting nerve roots and fibrous strands (Figures 3–5). On contrast-enhanced MRI, heterogeneous enhancement of the mass was demonstrated (Figure 6). A probable radiological diagnosis of neurofibroma/neuroblastoma was given.

## Treatment and follow-up

The mass was excised *via* a transabdominal extraperitoneal approach. Her symptoms were relieved, and on follow-up after 6 months, the patient was doing well. Histopathological examination revealed features of ganglioneuroma.

## Discussion

Ganglioneuromas are benign tumours of the sympathetic nervous system that originate from neural crest cells. They are considered to be a part of neuroblastic tumours, (neuroblastoma, ganglioneuroblastoma and ganglioneuroma) but are fully differentiated and benign. They can arise anywhere along the sympathetic nerve chain.^1^ The most common sites of occurrence of neuroblastic tumours are the posterior mediastinum, followed by the retroperitoneum, adrenal glands and neck. A presacral location is extremely rare. Only a few cases have been reported previously (< 20).^2^ It is usually seen in adolescents and young adults, with a slight female predominance.^3^

These tumours are grossly well-circumscribed, solid, encapsulated masses with presence of mature ganglion cells on microscopy. On imaging, they appear as well-circumscribed masses with calcifications in about 20% of cases. They appear as hypo- to isodense masses on CT scans. On MRI, they appear isointense on *T*_1_ and heterogeneous on *T*_2_ weighted images with low-to-moderate enhancement. Foraminal widening may be observed in these tumours, similar to changes observed in schwannomas and neurofibromas arising in this region, thus narrowing down the differential diagnosis in case of a presacral mass. It is difficult to differentiate from the other neural crest tumours. Ganglioneuromas may have punctate calcifications compared with coarse calcifications in neuroblastoma.^4^

The treatment is surgical excision. No additional radiotherapy or chemotherapy is required as it is a benign tumour and the patient usually does well on follow-up.

## Learning points

1. Although ganglioneuromas are rare in the sacral region, they should be considered as part of the differential diagnosis in people presenting with a pelvic mass as the prognosis is excellent compared with other neural crest tumours.
2. The imaging features such as extent, presence of foraminal widening and signal characteristics help us to narrow down the differentials and give an appropriate diagnosis.

## Consent

Informed consent has been taken from the patient's parents for publication of this case report.
